# Involvement of cellular metabolism in age-related LTP modifications in rat hippocampal slices

**DOI:** 10.18632/oncotarget.4188

**Published:** 2015-05-19

**Authors:** Dominika Drulis-Fajdasz, Tomasz Wójtowicz, Marcin Wawrzyniak, Jakub Wlodarczyk, Jerzy W. Mozrzymas, Dariusz Rakus

**Affiliations:** ^1^ Department of Animal Molecular Physiology, Institute of Experimental Biology, Wroclaw University, Wroclaw, Poland; ^2^ Laboratory of Neuroscience, Department of Biophysics, Wroclaw Medical University, Wroclaw, Poland; ^3^ Laboratory of Cell Biophysics, Department of Molecular and Cellular Neurobiology, Nencki Institute of Experimental Biology, Warsaw, Poland

**Keywords:** aging, plasticity, glycogen synthase, glycogen phosphorylase, dendritic spine maturation

## Abstract

Recent studies emphasized crucial role of astrocytic glycogen metabolism in regulation of synaptic transmission and plasticity in young animals. However, the interplay between age-related synaptic plasticity impairments and changes in energetic metabolism remains obscure. To address this issue, we investigated, in hippocampal slices of young (one month) and aged rats (20-22-months), the impact of glycogen degradation inhibition on LTP, mRNA expression for glycogen metabolism enzymes and morphology of dendritic spines. We show that, whereas in young hippocampi, inhibition of glycogen phosphorolysis disrupts the late phase of LTP in the Schaffer collateral-CA1 pathway, in aged rats, blockade of glycogen phosphorylase tends to enhance it. Gene expression for key energy metabolism enzymes, such as glycogen synthase and phosphorylase and glutamine synthetase showed marked differences between young and aged groups and changes in expression of these enzymes preceded plasticity phenomena. Interestingly, in the aged group, a prominent expression of these enzymes was found also in neurons. Concluding, we show that LTP in the considered pathway is differentially modulated by metabolic processes in young and aging animals, indicating a novel venue of studies aiming at preventing cognitive decline during aging.

## INTRODUCTION

Impaired synaptic plasticity is believed to be one of the major mechanisms underlying age-dependent cognitive decline [1]. The majority of excitatory transmission in the brain is mediated by glutamate-gated channels and it is widely accepted that decreased number, deficient trafficking and/or functional impairment of these receptors (especially of AMPA type) contribute to age-related plasticity decline [2]. In addition, it is known that synaptic plasticity phenomena are also manifested by profound alterations in spine shape [3, 4] but little is known how these structures change their propensity to morphological changes with age.

During the past decade a growing body of evidence has accumulated that astrocytes regulate properties of neuronal networks by supplying neurotransmitter precursors and by modulating neuronal signaling by e.g. releasing a broad spectrum of gliotransmitters [5]. Just recently it turned out that energetic metabolism of astrocytes is a crucial player in regulation of basal transmission and the plasticity phenomena in neuronal networks: the inhibition of glycogen breakdown and lactate release from astrocytes reduces the miniature excitatory postsynaptic currents in astrocyte-neuron co-cultures [6], inhibits memory consolidation in chicks [7] and disrupts memory formation in mice hippocampi [8]. However, it should be born in mind that slice or behavioral experiments [8, 9] were carried out on young or juvenile animals. Importantly, aging is known to affect the stability of long-term potentiation, LTP [10], a cellular mechanism of learning and memory (for review see in: [2]) and to alter the expression of energy metabolism enzymes in astrocytes [11]. However, the interplay between age-related synaptic plasticity impairments and changes in astrocyte energetic metabolism remains obscure. To address this issue, we have investigated, in hippocampal slices of young (one month) and aged rats (20-22-month), the impact of pharmacological blockade of glycogen degradation in astrocytes on LTP, mRNA expression for glycogen metabolism enzymes and the density and morphology of dendritic spines. Our results demonstrate that, in contrast to young hippocampi, inhibition of glycogen phosphorolysis elevates LTP magnitude in Schaffer collateral-CA1 (Sch-CA1) pathway in aged rats. The observed differences in the LTP amplitude and maintenance are accompanied by a different mode of dendritic spines maturation in young and aged animals. The results presented here also suggest that molecular changes in astrocytes energy metabolism precede plasticity phenomena, both in young and aged animals.

In conclusion, we show that LTP in the hippocampal Sch-CA1 pathway is differentially modulated by astrocytic metabolism in young and aging animals, indicating a novel venue of studies aiming at preventing memory problems and cognitive decline during aging.

## RESULTS

### Glycogen phosphorylase inhibitor oppositely influences LTP induction in young and aged rats

**Figure 1:**
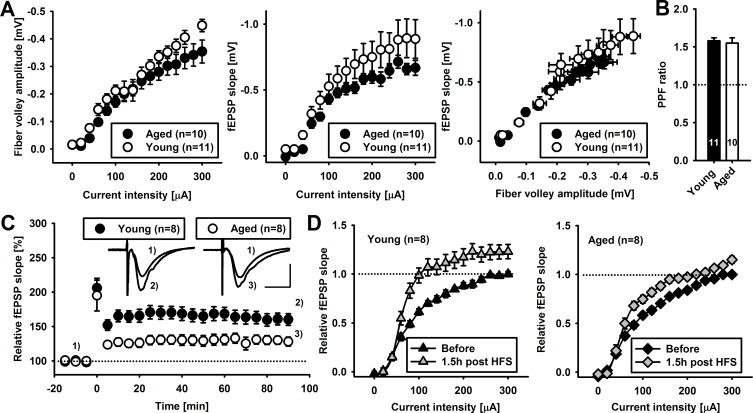
Acute hippocampal brain slices retain key properties during ageing **A.** Relationships between the mean fiber volley amplitude, fEPSP slope and stimulating current intensity in young (black circles) and aged (white circles) rats. Note that ageing does not significantly change basal properties of Sch-CA1 excitatory transmission. **B.** Average basal paired pulse facilitation ratio (50 ms gap) of fEPSP slope in young and aged animals. **C.** Average time-course of fEPSP slopes recorded in young (black circles) and aged animals (white circles) before and after tetanization (4 × 100 Hz, time 0 minutes) normalized to baseline values. Insets show exemplary fEPSPs recorded before (1) and 90 minutes (2,3) after tetanization (scale: 0.4 mV, 10 ms). Note an increased LTP magnitude in young compared to aged animals. **D.** Average fEPSP slopes plotted against current stimulus (input-output curve, I-O) before (black) and 90 minutes post tetanization (grey) in young (left panel) and aged animals (right panel). Note that tetanization resulted in larger left-ward shift in I-O curves in young compared to aged animals. Number in brackets depict number of slices compared.

Since it may be expected that young and aged animals could show major differences both in neuronal excitability in the selected pathway and in the synaptic transmission, these two aspects were investigated. Analysis of the ﬁber volley amplitude, which represents the number of stimulated axons ﬁring action potential, revealed no signiﬁcant difference between young and aged rats for the whole range of stimulating currents (0 - 300 μA; *F*_(2,335)_ = 0.462, *p* = 0.95; *n* = at least 10 slices, Figure 1A). To describe basal synaptic function in young and aged animals, we measured the I-O relationships for fEPSPs recorded in the stratum radiatum of CA1 region and elicited by stimulation of Schaeffer collaterals with increasing stimulus intensity. The slope of fEPSPs mirroring strength of basal glutamatergic synaptic transmission in acute brain slices of young animals was not different from that in aged animals (*F*_(2,335)_ = 0.36, *p* = 0.98; *n* = at least 10 slices, Figure 1A). Consequently, comparison of fEPSPs plotted against the ﬁber volley amplitude yielded analogous results (Figure 1A). Subsequently, we compared short–term synaptic plasticity in both groups by measuring the rate of fEPSP slope facilitation in response to paired stimulation (paired pulse facilitation, PPF; inter-stimulus interval 50 ms). We found no significant difference in PPF index in young compared to aged animal slices (PPF index was 1.58 ± 0.04 and 1.55 ± 0.07 in young and aged animals, respectively, *n* = at least 10 slices, *p* = 0.64, Figure 1B).

Next, we assessed the impact of age on LTP characteristics. To this end we applied high frequency stimulation (HFS, 4 × 100 Hz), setting the stimulation current at the value yielding approx. 30 - 40 % max. slope of fEPSP before HFS. In young animals, HFS resulted in a post-tetanic potentiation (PTP) that reached 206.1 ± 14.07 % of basal fEPSP slope (*n* = 8 slices, *p* < 0.01 compared to baseline, Figure 1C) which subsequently declined reaching a new steady value of 160.2 ± 8.45 % of the baseline at 90 minutes after LTP induction (*n* = 8, *p* < 0.01 compared to baseline, Figure 1C). In aged animals, average PTP was 195.06 ± 22.39 % of basal signal (*n* = 8, *P* < 0.01 compared to baseline but not statistically different from PTP in young animals, *p* = 0.22) after which the signal decreased and stabilized attaining the value of 126.8 ± 7.3 % of basal fEPSP slope (90 min post HFS, *n* = 8, *p* < 0.01 of baseline, Figure 1C). Following PTP, the magnitude of fEPSPs potentiation recorded in young animals was significantly larger than in aged animals throughout the time of recording (*p* < 0.001 for time window of 5-90 minutes, unpaired student *t*-test, Figure 1C). In young animals, I-O relationship for fEPSP slope analyzed 90 min. post LTP induction revealed a significant upward shift (*F*_(2,255)_ = 4.137, *p <* 0.001, Figure 1D). Similarly, in aged animals, upward shift was also observed (*F*_(2,255)_ = 1.703, *p <* 0.05, Figure 1D). We additionally monitored PPF ratio 90 minutes post LTP induction and compared its value with those recorded before LTP in basal conditions. In slices from young animals, LTP magnitude at 90 minutes post induction significantly correlated with reduction of PPF ratio (relative PPF change was 0.90 ± 0.019, *n* = 8, Pearson correlation coefficient −0.628, *p* < 0.01, data not shown). Similarly, in aged animals, LTP magnitude correlated with PPF ratio reduction (relative PPF change was 0.91 ± 0.04, *n* = 8, Pearson correlation coefficient −0.669, *p* < 0.05, data not shown). In conclusion, slices of both young and aged animals did not significantly differ in viability and basal synaptic transmission within considered time window but the extent of LTP was significantly smaller in aged group for most of the recording time. Moreover, successful induction of LTP correlated with respective shift in I-O curves and PPF reduction.

We next checked the impact of glycogen phosphorylase blockade (5 μM BAY) on synaptic transmission in the Sch-CA1 pathway in young and aged animals. We found that thirty to ninety minutes of drug application did not significantly affect fEPSP slope recorded in slices from both young and aged animals upon basal stimulation (fEPSP relative change was 0.98 ± 0.01 and 0.99 ± 0.02, respectively, *n* = 8, *p* > 0.7, data not shown). Moreover, PPF ratio in the presence of BAY was not altered in both groups investigated (PPF ratio was 1.53 ± 0.05 and 1.46 ± 0.04 in slices of young and aged animals, respectively; *n* = 8, *p* = 0.47 and *p* = 0.314 compared to respective controls, data not shown). Thus, BAY did not affect basal synaptic transmission or short-term facilitation.

**Figure 2:**
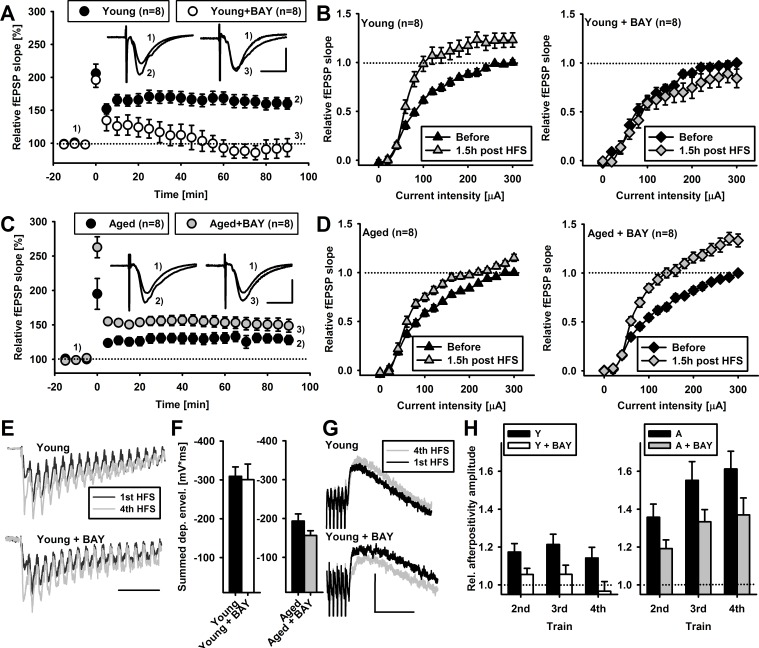
Glycogen phosphorylase inhibitor, BAY U6751, differentially affects LTP in young and aged animals **A.** Average time-course of fEPSP slopes recorded in young animals in the absence (black circles) or presence of bath applied inhibitor (white circles) before and after tetanization (4 × 100 Hz, time 0 minutes) normalized to baseline values. Insets show exemplary fEPSPs recorded before (1) and 90 minutes (2,3) after tetanization (scale: 0.4 mV, 10 ms). Note that bath application of BAY (10 μM) completely abolished LTP in young animals. **B.** Average fEPSP slopes plotted against current stimulus (I-O curves) before (black) and 90 minutes post tetanization (grey) in young animals in the absence (left panel) or presence of inhibitor (right panel). Note that BAY application completely abolished left-ward shift of I-O curve observed in controls. **C.-D.** The same experiments presented in A-B performed in slices of aged animals. Note that bath application of BAY did not abolish but rather enchanced LTP magnitude in aged animals (C). Note that bath application of BAY enchanced left-ward shift of I-O curve compared to aged controls. **E.** Exemplary traces of initial fEPSPs recorded in young animals during first (black trace) and fourth (grey trace) high frequency stimulation (HFS, 100 Hz) in the absence (upper panel) and in the presence of bath applied inhibitor (lower panel). Note that BAY does not significantly alter the time-course of fEPSPs during HFS (scale: 50 ms, amplitudes were normalized). **F.** Average depolarization envelopes for all four trains of stimuli in young (left panel) and aged (right panel) animals. Note that in the presence of inhibitor (BAY), average depolarization envelopes did not significantly change. **G.** Exemplary traces of fEPSPs time-course recorded immediately following cessation of HFS in young animals (black traces). Note the positive deflection occurring immediately following HFS (afterpositivity, AFP). Panels show exemplary trace of AFP recorded following first (black trace) and fourth (grey trace) in the absence (upper panel) and in the presence (lower panel) of the inhibitor (scale: 0.15 mV, 100 ms). **H.** Average AFP amplitude following subsequent trains of HFS normalized to AFP of the first train in young (left panel) and aged animals (right panel). Note that BAY application significantly reduced the scaling of AFP amplitude in both young and aged animals (*P* < 0.05, ANOVA). Number in brackets depict number of slices compared.

We then considered the impact of BAY on synaptic plasticity. In young animals, HFS applied in the presence of BAY resulted in similar PTP (195.9 ± 11.1 % of baseline, *n* = 8, *p* = 0.1 compared to untreated controls) but LTP maintenance phase was completely disrupted (LTP at 90 minutes post induction was 93.3 ± 13.6 % of baseline, *p* < 0.01 compared to untreated controls, *n* = 8 slices, Figure 2A). Moreover, I-O curves generated before and 90 minutes post LTP induction in BAY treated slices were not significantly shifted (*F*_(2,256)_ = 0.340, *p =* 0.99, Figure 2B, significantly different compared to I-O curves in control post LTP *F*_(2,256)_ = 2.896, p < 0.001) and PPF ratio, following HFS, did not significantly change (relative PPF ratio change was 1.03 ± 0.02, *p* = 0.29, data not shown).

Next we repeated this experimental paradigm for slices from aged animals. Surprisingly, we found that in the presence of BAY, PTP was significantly larger than in control aged group (fEPSPs slope potentiation was 262.69 ± 15.2 % of basal signal, *p* < 0.05 relative to aged controls, *n* = 8, Figure 2C). Following PTP, the magnitude of fEPSPs potentiation recorded in aged animals in the presence of BAY was significantly larger than in aged controls in the first 60 minutes following HFS (*p* < 0.001, unpaired student *t*-test, Figure 2C). After that time LTP magnitude in the presence of BAY, although on average larger, was not significantly different from that recorded in aged controls (65-90 min., p > 0.05, Figure 2C). In the same set of preparations, we observed a significant upward shift in I-O relationship for fEPSP slope analyzed 90 min post LTP (*F*_(2,256)_ = 2.274, *p =* 0.004, Figure 2D). This upward shift was significantly larger than that observed in control slices of aged animals (*F*_(2,256)_ = 1.450, *p =* 0.045, most evident for strong stimuli of 200μA-300μA, Figure 2D). In slices from aged animals treated with BAY, basal PPF ratio was 1.46 ± 0.04 and was not significantly different from the respective control (*p* = 0.314, *n* = 8, data not shown). After LTP induction, average PPF ratio was reduced to similar extent as in controls (relative PPF ratio change 90 minutes post HFS was 0.91 ± 0.03, *p* = 0.89 compared to controls, data not shown). Altogether, unlike young animals, pretreatment of slices from aged animals with phosphorylase inhibitor BAY did not interfere with the magnitude of LTP in Sch-CA1 projection showing even a trend to increase it.

Divergent effects of BAY administration on post-tetanic potentiation and, subsequently, on LTP magnitude in young and aged animals could indicate altered conditions of LTP induction. To get an insight into phenomena occurring upon stimulation, we next analyzed fEPSPs during trains of HFS. In slices from young animals, average depolarizing fEPSP envelope during the 1^st^ train was −55.33 ± 3.86 mV*ms and it was not changed upon bath application of the drug (−52.88 ± 5.79 mV*ms, *p* = 0.7, *n* = 8, data not shown). Summed depolarizing envelope following four, one second long, HFS trains was −309.2 ± 24.1 mV*ms, *n* = 8, Figure 2F), and this parameter was not changed upon bath application of BAY (−300.45 ± 40.3 mV*ms, *n* = 8, *p* = 0.72, Figure 2F). Analogous analysis performed on data from aged animals revealed that average depolarizing fEPSP envelope during the 1^st^ train was significantly smaller compared to young animals (−40.1 ± 4.2 mV*ms, *n* = 8, *p* = 0.019 compared to young animals) and it was not changed upon bath application of BAY (−37.47 ± 4.81 mV*ms, *p* = 0.7, *n* = 8, data not shown). Summed depolarizing envelope following four HFS trains was −193.4 ± 18.4 mV*ms, *n* = 8, Figure 2F), and this parameter was not significantly different upon bath application of BAY (−156.3 ± 12.3 mV*ms, *n* = 8, *p* = 0.09, Figure 2F).

Next we analyzed the amplitude of afterpositivity (AFP) that peaked approximately 50 – 100 ms after the cessation of HFS (Figure 2G). In slices of young animals, average afterpositivity amplitude occurring following 1^st^ HFS was 0.19 ± 0.01 mV (*n* = 8) and it was not different in slices treated with BAY (0.23 ± 0.02, *n* = 8, *p* = 0.22, data not shown). Following repeated HFS stimulation, afterpositivity amplitude varied with each subsequent HFS (relative AFP amplitude normalized to 1^st^ train value was 1.17 ± 0.04, 1.21 ± 0.05 and 1.14 ± 0.05 for 2^nd^ - 4^th^ train, respectively, *n* = 8, Figure 2H). In the presence of BAY, the increment of AFP amplitude was significantly attenuated (relative AFP amplitude in the presence of BAY was 1.05 ± 0.03, 1.05 ± 0.04 and 0.96 ± 0.05 for 2^nd^ - 4^th^ train, respectively, *F*_(1,48)_ = 14.326, *n* = 8, *p* < 0.001 compared to untreated controls, Figure 2H).

In slices of aged animals, average afterpositivity amplitude occurring following 1^st^ HFS was 0.17 ± 0.02 mV (*n* = 8, not significantly different from slices of young animals, *p* = 0.28) and it was not different in slices treated with BAY (0.16 ± 0.01, *n* = 8, *p* = 0.69, student *t*-test, data not shown). With each subsequent HFS train, relative AFP amplitude increased (relative afterpositivity amplitude was 1.35 ± 0.06, 1.55 ± 0.09 and 1.61 ± 0.09 following 2^nd^ - 4^th^ train, respectively, *n* = 8, Figure 2H). In the presence of BAY, the increment of AFP amplitude was significantly attenuated (relative afterpositivity amplitude in the presence of BAY was 1.19 ± 0.04, 1.33 ± 0.06 and 1.36 ± 0.08 for 2^nd^ - 4^th^ train, respectively, *F*_(1,48)_ = 6.857, *n* = 8, *p* = 0.01 compared to untreated controls, Figure 2H). In conclusion, BAY had no impact on depolarizing envelope during LTP induction, but attenuated HFS-induced changes in afterpositivity amplitude in both young and aged groups.

### mRNA expression profile depends on the electrophysiological stimulation and the animal age

**Figure 3:**
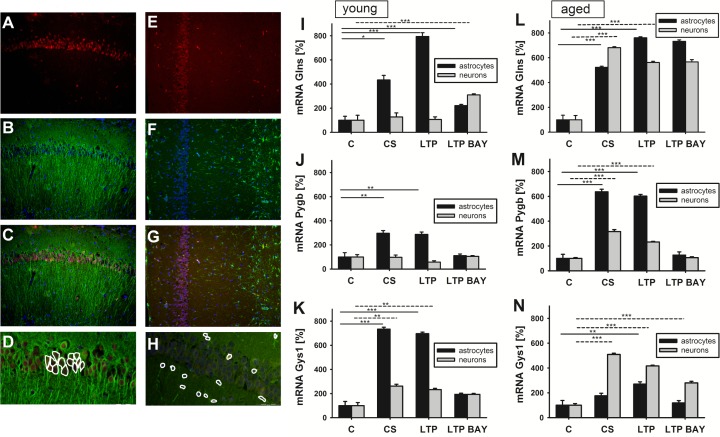
Effect of glycogen phosphorylase (Pygb) inhibition on mRNA expression of key metabolic enzymes following LTP depends on cell type and age **A.-H.** Localization of mRNA for Pygb in hippocampal slices of young rats 90 minutes following LTP induction. **A.** and **E.** FISH staining against Pygb; **B.** and **F.** IF staining against MAP-2 (B) or GFAP (F); **C.** merged A+B; **G.** merged E+F. **D.** exemplary analysis of a population of pyramidal cells (CA1 hippocampal region) or astrocytes (H) with marked region of mRNA expression analysis. **I.-N.** Statistics of the effect of glycogen phosphorylase inhibition with BAY U6751 on the basal and HFS stimulation-induced changes in the expression of mRNA for glutamine synthetase (Glns), Pygb or glycogens synthase 1 (Gys1) in young (I-K) or aged (L-N) rat hippocampi. mRNA expression analysis performed for basal stimulated (CS), LTP-induced (LTP) and LTP-induced in the presence of BAY (LTP BAY) samples. The expression in unstimulated control (C) was normalized to 100 %. Average number of analyzed cells in a probe was *n* = 2282 and *n* = 1049 for astrocytes and neurons, respectively. The *p* values (asterisks) for astrocytes (black bars) and neurons (grey bars) were as follows: I) CS *vs* C - 4.92E-02 and 0.64; LTP *vs* C - 1.34E-02 and 0.95; LTP BAY *vs* C - 4.63E-05 and 2.59E-05. J) mRNA for Pygb: CS *vs* C - 9.82E-03 and 0.9; LTP *vs* C - 7.20E-03 and 5.07E-02; LTP BAY *vs* C - 0.75 and 0.85. K) mRNA for Gys1: CS *vs* C - 9.47E-06 and 1.59E-03; LTP *vs* C - 2.88E-07 and 8.48E-04; LTP BAY *vs* C - 5.00E-04 and 3.97E-02; L-N for aged rats L) mRNA for Glns: CS *vs* C - 4.03E-08 and 4.64E-08; LTP *vs* C - 1.32E-09 and 4.48E-06; LTP BAY *vs* C - 1.61E-07 and 6.73E-04. M) mRNA for Pygb: CS *vs* C - 3.51E-03 and 7.35E-04; LTP *vs* C - 3.3E-0402 and 2.24E-04; LTP BAY *vs* C - 0.71 and 0.8. N) mRNA for Gys1: CS *vs* C - 0.14 and 1.07E-05; LTP *vs* C - 9.56E-03 and 1.06E-07; LTP BAY *vs* C - 0.67 and 2.47E-04.

In slices that were subjected to electrophysiology recordings, we checked the correlation between neuronal activity and the mRNA expression for several metabolic enzymes in neurons and astrocytes (*n* indicates number of analyzed individual cells, see Materials and Methods; Figure 3). As a reference gene we used glutamine synthetase (*Glns*), an astrocyte-specific enzyme, which catalyzes the condensation of glutamate and ammonia to form glutamine. Gln protein in neurons is practically undetectable and its expression is activated only in absence of astrocyte e.g. in. pure neuronal culture [12]. We found that 90 minutes after LTP induction, astrocytic mRNA for *Glns* significantly increased in both young and aged animals (respectively 794 % ± 31, *n* = 2040 and 760 % ± 8, *n* = 1965 compared to non-stimulated tissue samples, *p* < 0.01, Figure 3I, 3L). Moreover, the basal (90min, 0.1 Hz) exogenous stimulation also led to increase in mRNA for *Glns* in astrocytes (433 % ± 38, *n* = 2505 and 523 % ± 9, *n* = 2011 for young and aged rats respectively). Simultaneously, we could not observe any increase in mRNA for *Glns* in young rat's neurons. It is noteworthy that the level of *Glns* mRNA after exogenous stimulation was significantly higher in neurons of aged rats compared to the young animals (Figure 3I *vs* 3L).

Next, we checked the mRNA expression for brain isozyme of glycogen phosphorylase (Pygb) and muscle isoform of glycogen synthase (Gys1) and we found that basal stimulation (90min, 0.1 Hz) significantly elevated mRNA expression for both enzymes in astrocytes (in young as well as in aged rats) as compared to unstimulated hippocampal sections but in the case of neurons the increase was restricted only to the aged animals (Figure 3J-3K and Figure 3M-3N). Evidently, exogenous stimulation of the Sch-CA1 pathway resulted in statistically significant increase in mRNA level for key glycogen metabolism enzymes in astrocytes.

Interestingly, we found that even basal stimulation (90min, 0.1 Hz) of the slices was sufficient for the increase of *Pygb* and *Gys1* level in astrocytes (296 % ± 22, *n* = 3101 and 734 % ± 16, *n* = 2682 respectively for *Pygb* and *Gys1* in young rats and 636 % ± 21, *n* = 2184 and 176 % ± 20, *n* = 2447 respectively for *Pygb* and *Gys1* in aged rats). Further stimulation using 4 × 100 Hz train did not strengthen the effect on mRNA level in young and aged astrocytes (287 % ± 19, *n* = 2253 and 696 % ± 13, *n* = 2747 respectively for *Pygb* and *Gys1* in young rats; 602 % ± 13, *n* = 2858 and 270 % ± 18, *n* = 1908 respectively for *Pygb* and *Gys1* in aged animals, Figure 3J-3K and Figure 3M-3N). As it is shown in Figure 3, mRNA up-regulation for key glycogen metabolism enzymes occurred in astrocytes in both age groups. On the other hand, the changes in the mRNAs expression in neurons showed a different pattern. In young animals, we observed relatively constant level of *Pygb* in neurons regardless of electrophysiological treatment (C *vs* CS *vs* LTP, Figure 3J) whereas, for Gys1 we found a doubling of mRNA expression following basal stimulation and LTP induction as compared to its initial level in hippocampal slices (CS *vs* C = 261 % ± 15, LTP *vs* C = 232 % ± 10, Figure 3K). In contrast to young rats, neurons of aged animals exhibited significantly higher levels of mRNA for Pygb in CS (316 % ± 15, n = 1157) and LTP slices (231 % ± 5, *n* = 1314 see Figure 3M) and for Gys1 as well (CS *vs* C = 509 % ± 10; LTP *vs* C = 416 % ± 6, Figure 3N).

In conclusion, mRNA level for key metabolic enzymes (Gys1, Pygb and Glns) is differentially regulated by exogenously evoked synaptic activity in Sch-CA1 region of young and aged rat hippocampal slices.

### Impact of glycogen phosphorylase inhibitor on mRNA expression level of metabolic enzymes

Next, we repeated the above described FISH analysis in slices in which LTP was evoked in the presence of glycogen phosphorylase inhibitor BAY. With regard to Glns mRNA expression, we found that in astrocytes of young rats, mRNA level of this enzyme was significantly lower compared to LTP slices in the absence of the drug (LTP = 794 % ± 31, *n* = 2040 *vs* LTPBAY = 221 % ± 10, *n* = 1519 Figure 3I). In contrast, BAY treatment had no impact on Glns mRNA expression in astrocytes or neurons in aged animals (LTP BAY astrocytes = 732 % ± 11, *n* = 1193 and LTP BAY neurons = 566 % ± 18, *n* = 1212, Figure 3L). Next, we investigated the mRNA expression for Pygb and Gys1. We found that treatment of slices with BAY prevented the up-regulation of mRNA expression for Pygb and Gys1 in astrocytes and neurons of both young and aged animals (Figure 3J, 3M and Figure 3K, 3N). Thus, in the presence of glycogen phosphorylase inhibitor, mRNA up-regulation following LTP induction is lost regardless of magnitude of LTP (see Figure 2A and 2C)

### Beta amyloid spots are absent in young and appear in tissue of aged animals

**Figure 4:**
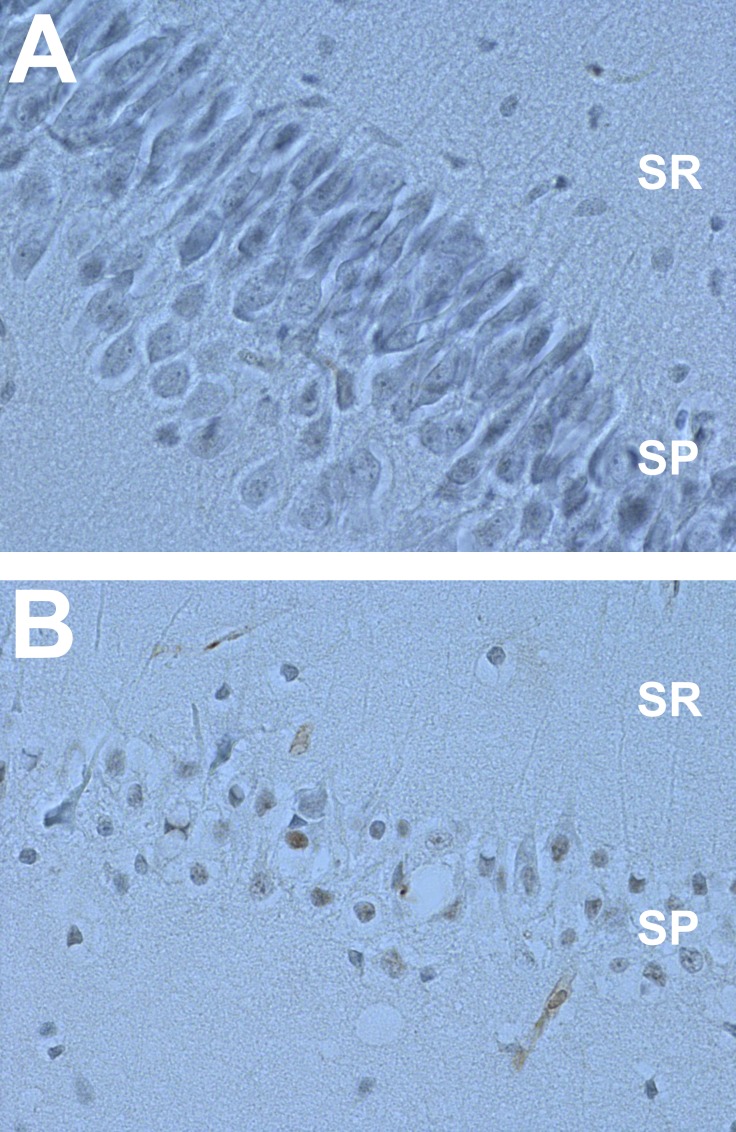
Aging-related changes in hippocampal area are associated with the appearance of beta amyloid spots and distorted geometry of neuronal network Immunochemical staining against beta-amyloid peptide in CA1 hipocampal region of **A.** young rat and **B.** aged rat. SR, stratum radiatum; SP, stratum piramidale.

To see age-related changes in the structure of hippocampal region we stained the slices using antibodies against beta-amyloid peptide for both age groups of animals (n for each = 4). We found that in the considered here Sch-CA1 projection, the beta-amyloid peptide was present in all slices from aged animals hippocampi and was virtually absent in those from young rats. Slightly unexpectedly, in the former group, it was localized mainly inside cells and, to a lesser extent, in the extracellular space. Moreover, the slices from aged animals showed reduced neuron density and distorted geometry of hippocampal area as compared to the young ones (Figure 4).

**Table 1:** Cy3 fluorescence probes used for FISH analysis

	enzyme	Protein (gene) symbol	Oligonucleotide sequence anti-mRNA 5′-3′
1	glycogen synthase type 1	Gys1 (Gys1)	GGGTCCCCCCTCGATCAGCCAACGCCCAAAATACACCTTA
2	glycogen phosphorylase brain	Pygb (Pygb)	AGGGTGGCGGCCACCACAAAGTACTCCTGCTTCAGCCGCA
3	glutamine synthetase	Glns (Glns)	GCTTGATGCCTTTGTTCAAGTGGGAACTTGCTGAGGTGGC

**Table 2:** Categories of hippocampal slices prepared in electrophysiology experiments for the dendritic spine morphological analysis

hippocampal slice name	abbreviation	electrophysiological stimulation
C	Control in ACSF buffer	No
C BAY	Control in ACSF buffer+5uM BAY	No
CS 30	ACSF buffer	30 min of basal stimulation
LTP 30	ACSF buffer	30 min after LTP induction
CS BAY 30	ACSF buffer+5uM BAY	30 min of basal stimulation with BAY
LTP BAY 30	ACSF buffer+5uM BAY	30 min after LTP induction with BAY
CS 90	ACSF buffer	90 min of basal stimulation
LTP 90	ACSF buffer	90 min after LTP induction
CS BAY 90	ACSF buffer+5uM BAY	90 min of basal stimulation with BAY
LTP BAY 90	ACSF buffer+5uM BAY	90 min after LTP induction with BAY

### The effect of plasticity induction, glycogen phosphorylase inhibition and aging, on dendritic spines morphology

In order to investigate age dependent effect of glycogen phosphorylase blockade on structural plasticity of dendritic spines we analyzed dendritic spine morphology in secondary/ternary dendrites of CA1 hippocampal region. To this end, neurons were stained with a lipophilic dye (DiI) then length and head width of dendritic spine were measured. Thus we were able to calculate further a scale-free parameter length-to-width ratio, which effectively describes the spine shape.

First, to check whether BAY treatment affects the morphological parameters of dendritic spines or whether the basal stimulation influence the spine shape characteristic, we did a number of comparisons between control (unstimulated) samples (Table 3). In none of mentioned juxtapositions we found any difference between animal groups in terms of BAY presence and basal stimulation (Table 3). Thus we conclude that all analyzed animal groups were equivalent for further analysis of HFS induced changes in spine morphology.

**Table 3:** Dendritic spine morphology of young and aged rats Comparison of unstimulated samples at time zero (C) and control slices after 30 min and 90 min of basal stimulation (CS 30, CS 90). The BAY treatment and the basal stimulation did not induce differences between dendritic spine shape in analysed groups (C BAY, CS BAY 30, CS BAY 90).

increase	↑	**young**	increase	↑	**aged**
constants	↔		constants	↔	
decrease	↓		decrease	↓	
**Effect of BAY** **treatment**		length/ width ratio	**Effect of BAY** **treatment**		length/ width ratio
C (n=12 slices) vs C BAY (n=14 slices)		↔ p=0.420	C (n=14 slices) vs C BAY (n=17 slices)		↔ p=0.207
CS 30 (n=6 slices) vs CS BAY 30 (n=14 slices)		↔ p=0.131	CS 30 (n=11 slices) vs CS BAY 30 (n=12 slices)		↔ p=0.771
CS 90 (n=7 slices) vs CS BAY 90 (n=12 slices)		↔ p=0.772	CS 90 (n=15 slices) vs CS BAY 90 (n=19 slices)		↔ p=0.995
**Effect of** **basal stimulation**			**Effect of** **basal stimulation**		
C (n= 12 slices) vs CS 30 (n=6 slices)		↔ p=0.570	C (n=14 slices) vs CS 30 (n=11 slices)		↔ p=0.363
C (n= 12 slices) vs CS 90 (n=7 slices)		↔ p=0.804	C (n=14 slices) vs CS 90 (n=15 slices)		↔ p=0.167

It is well established that LTP induction alters spine morphology. Since, as shown herein, the extent of LTP was markedly affected by Pygb blockade (by BAY) in an age-dependent manner, we were seeking to find a correlation between these functional data and dendritic spine morphology. In order to assess the impact of BAY on dendritic spines after LTP induction, we compared spine geometry at 30 min and 90 min after HFS stimulation *vs* control groups (basal stimulation at the same time points). Major trends observed based on results of our analysis are summarized in the Table 4.

**Table 4:** Effect of BAY on dendritic spine morphology, of young and aged rats, upon HFS Comparison of basal stimulated control and LTP induced slices at parallel time point of electrophysiological stimulation (after 30 min and 90 min - CS 30, LTP 30 and CS 90, LTP 90). Spines of both, young and aged rats changed after HFS stimulation. Morphological analysis indicates the dendritic spine maturation i.e. spines become mushroom shaped, with lower values of length-to-width ratio.

increase	↑	**young**	increase	↑	**aged**
constants	↔		constants	↔	
decrease	↓		decrease	↓	
**Effect of HFS** **stimulation**		length/ width ratio	**Effect of HFS** **stimulation**		length/ width ratio
CS 30 (n=6 slices) vs LTP 30 (n=7 slices)		↓ p=0.002	CS 30 (n=11 slices) vs LTP 30 (n=12 slices)		↓ p<0.0001
CS BAY 30 (n=14 slices) vs LTP BAY 30 (n=13 slices)		↓ p<0.0001	CS BAY 30 (n=12 slices) vs LTP BAY 30 (n=24 slices)		↓ p<0.0001
	
CS 90 (n=7 slices) vs LTP 90 (n=12 slices)		↓ p<0.0001	CS 90 (n=15 slices) vs LTP 90 (n=8 slices)		↓ p<0.0001
CS BAY 90 (n=12 slices) vs LTP BAY 90 (n=11 slices)		↓ p<0.0001	CS BAY 90 (n=19 slices) vs LTP BAY 90 (n=22 slices)		↓ p<0.0001

We analyzed length-to-width ratio (i.e. the length divided by the width), which reflects the spine shape and thus effectively describes the spine form. Decrease in these parameters shows change in shapes towards being shorter and wider, thus being more like mushroom spines that are indicated as more matured. We showed statistically significant decrease in the length-to-width parameter after LTP in both young and aged rats CS 90 *vs* LTP 90 (young: 2.87 ± 0.36 (*n* = 7 slices) *vs* 1.84 ± 0.24 (*n* = 12 slices), p-value t-test, *p* < 0.005, aged: 3.30 ± 0.51 (*n* = 15 slices) *vs* 2.35 ± 0.28 (*n* = 8 slices), *p*-value t-test *p* < 0.005). Similar differences in the length-to-width parameter of spines occurred in presence of BAY in both young and aged rats CS BAY 90 *vs* LTP BAY 90 (young: 2.92 ± 0.32 (*n* = 12 slices) *vs* 2.21 ± 0.34 (*n* = 11 slices), *p*-value *t*-test, *p* < 0.005, aged: 3.30 ± 0.47 (*n* = 19 slices) *vs* 2.43 ± 0.26 (*n* = 22 slices), *p*-value *t*-test *p* < 0.005).

**Figure 5:**
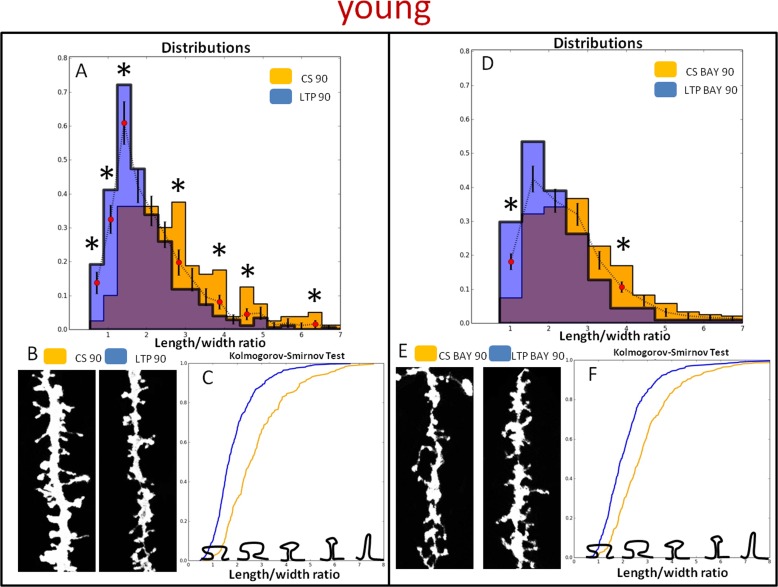
Glycogen phosphorylase inhibitor, BAY U6751, affects HFS induced dendritic spine morphology in young animals **A.** and **D.** Distribution of dendritic spines shape parameter (length-to-width) in LTP-induced (LTP 90) and LTP-induced in the presence of BAY (LTP BAY 90) samples in respect to the basal stimulated controls at 90 min (CS 90 and CS BAY 90 respectively). (*) indicates bins with statistically significant differences. **B.** and **E.** Examples of DiI-stained neurons in the CA1 area of rat hippocampus (pictures present secondary apical dendrites). **C.** and **F.** Cumulative frequency of the shape parameter (length/width) of spines in control and after LTP induction in brain slices. Kolmogorov-Smirnov test revealed significant differences in the spine shape parameters of CS 90 -compared with LTP 90 and CS BAY 90 compared with LTP BAY 90 (*p* < 0.001).

**Figure 6:**
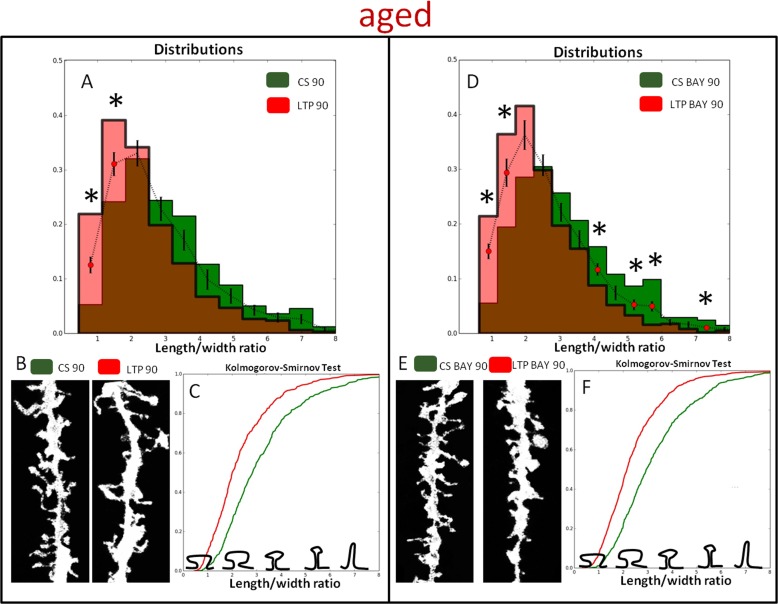
Glycogen phosphorylase inhibitor, BAY U6751, affects HFS induced dendritic spine morphology in aged animals **A.** and **D.** Distribution of dendritic spines shape parameter (length-to-width) in LTP-induced (LTP 90) and LTP-induced in the presence of BAY (LTP BAY 90) samples in respect to the basal stimulated controls at 90 min (CS 90 and CS BAY 90 respectively). (*) indicates bins with statistically significant differences. **B.** and **E.** Examples of DiI-stained neurons in the CA1 area of rat hippocampus (pictures present secondary apical dendrites). **C.** and **F.** Cumulative frequency of the shape parameter (length/width) of spines in control and after LTP induction in brain slices. Kolmogorov-Smirnov test revealed significant differences in the spine shape parameters of CS 90 -compared with LTP 90 and CS BAY 90 compared with LTP BAY 90 (*p* < 0.001).

Because the individual subpopulations of spines might determine the structural plasticity upon HFS we divided the spines into subpopulations according to the length-to-width parameter (Figures 5A, 5D and 6A, 6D). To provide a detailed description of the spine shape dynamics upon stimulation we analyzed age-dependent changes of individual bins of length-to-width distribution both in the presence and absence of BAY. This approach led to the identification of spine groups that displayed different dynamics of morphological changes. We found that BAY treatment restrained HFS-induced maturation of spines in young rats. The statistically significant differences were found solely in two bins of the distribution (Figure 5D). More prominent changes of shape parameters (toward mushroom shaped spines i.e. lower values of length-to-width) were observed in spines of aged rats (Figure 6D). The analysis of spine shape (Figures 5B, 5E and 6B, 6E) and cumulative distributions of shape parameter (Figures 5C, 5F and 6C, 6F) further support this observation (cf. Figure 5F and Figure 6F). The maximum difference (D) between cumulative curves for young and aged animals were found to be: D = 0.26 for CS 90 *vs* LTP 90, D = 0.26 for CS BAY 90 *vs* LTP BAY 90 and D = 0.39 for CS 90 *vs* LTP 90, D = 0.29 for CS BAY 90 *vs* LTP BAY 90, respectively.

Finally, we analyzed the aged dependent effect of inhibition of glycogen degradation on density of dendritic spines of interest. The incubation with BAY did not affect the total density of dendritic spines upon HFS induced LTP (Table 5).

**Table 5:** The mean density of spines in analysed groups of rats (data from 90 min of stimulation) CS - basal stimulated control; LTP - highfrequency stimulated slices; BAY - indicate the inhibitor presence during experiment.

slice group	young	aged
LTP 90	1.13 ± 0.13 μm^−1^	1.10 ± 0.13 μm^−1^
LTP BAY 90	1.09 ± 0.18 μm^−1^	1.10 ± 0.10 μm^−1^
CS 90	1.16 ± 0.20 μm^−1^	1.01 ± 0.06 μm^−1^
CS BAY 90	1.08 ± 0.16 μm^−1^	0.98 ± 0.09 μm^−1^

## DISCUSSION

In this paper we demonstrate that the inhibition of glycogen breakdown in astrocytes has an opposite effect on the LTP magnitude in young and aged rats, in young group it disrupts LTP maintenance whereas in the aged ones it appears to favor elevated extent of LTP. Importantly, alteration in LTP dependence on glycogen phosphorolysis in young and aged groups are corroborated by observation of different modes of dendritic spines maturation in young and aged animals (Figure 5, 6). Evidently, the inhibition of glycogen degradation suppressed HFS-induced spine maturation in young animals, whereas the opposite was seen in aged rats. The most intriguing question emerging from these findings concerns age-related changes in the astrocytic metabolism supporting the synaptic plasticity. Although, lactate produced by astrocytes from extracellular glucose is known to be the main energy substrate for neurons [13, 14], it was proposed that glycogen-derived, but not glucose-derived, lactate is a prerequisite for the plasticity phenomena in neonatal and young animals [15]. In agreement with this scenario, it was also shown that inhibition of glycogen breakdown and lactate release from astrocytes disrupted memory formation in hippocampus [8, 16] and impaired LTP [8]. It seems thus that indeed, a key to understand the mechanism underlying our observations lies in differential roles of glycogen phosphorolysis and their consequences in neuronal-astrocytic cross-talk. Importantly, upon basal stimulation (BS), there were no differences between fEPSPs measured in aged and young groups and, in these conditions, BAY had no effect. Thus, glycogen phosphorolysis appears to affect mainly the synaptic plasticity rather than the basal activity. Depolarizing envelope measured during LTP induction was not affected by BAY but BAY treatment significantly attenuated relative changes in afterpositivity (AFP) amplitude following HFS in both age groups. Afterpositivity occurring following intense neuronal activity is believed to primarily mirror activity of postsynaptic calcium- and voltage-dependent potassium conductances (e.g [17-20]) which play an important role in LTP, learning and memory [21-23]. Decreased dynamics of AFP in subsequent HFS trains may reflect a decreased Ca^2+^ entry upon BAY treatment. It is possible that changes in AFP result from BAY-induced drop in extracellular lactate but the precise mechanism remains to be determined. It should be kept in mind that L-lactate may serve also signaling functions that are not exclusively related to energy metabolism. For instance, lactate may act as a signaling molecule enhancing the acid-sensing Na^+^ channel [24] and evoking excitation in locus coeruleus [25]. Although previous studies have demonstrated that application of exogenous L-lactate is sufficient to support LTP in hippocampus formation [8, 16, 26], the experiments recently performed in our laboratory revealed that L-lactate was able to support LTP in Sch-CA1 pathway only in young but not aged animals (unpublished data). In addition, it is not entirely clear to what extent the impact of BAY concerns pre- or postsynaptic mechanisms. Importantly, long-term BAY treatment did not affect the basal transmission or short term facilitation assessed as PPF (Figure 1B) in young and aged rats, indicating the lack of effect on presynaptic mechanisms under these conditions. However, whereas BAY had no effect on PTP in young animals, in the aged group, PTP was increased in the presence of this compound. This observation suggests that differences in mechanisms of BAY effect on LTP in the two groups may involve some presynaptic mechanisms. Moreover, in both young and aged group, HFS led to a significant reduction of PPF but BAY did not influence this effect. In general, changes in short term plasticity parameters such as PPF or PTP may indicate a presynaptic change (e.g. [27, 28] and in considered Sch-CA1 pathway, a presynaptic component of LTP has been described [29] and therefore a presynaptic BAY action cannot be excluded. On the other hand, attenuation of PPF following LTP induction could also result from altered responsiveness of post-synaptic receptors due to post-synaptic calcium signaling [30, 31]. It seems thus that differential impact of BAY on late LTP phases in young and old groups appears most consistent with postsynaptic effects while the contribution from presynaptic mechanisms remain to be established.

Intriguingly, basal and HFS stimulation affected the abundance of mRNA for glycogen metabolism enzymes. The mRNA level for all studied enzymes in astrocytes increased in both age groups after BS. In the case of young animals, HFS stimulation did not cause any further increase of mRNA amount for *Gys1 and Pygb*. However, application of BAY precluded stable LTP maintenance (Figure 2) and in these conditions expression of *Glns*, *Pygb* and *Gys1* in astrocytes (after HFS) was smaller than in controls (Figure 3). This suggests that BS is able to induce gene expression for key metabolic enzymes which appears to be a preparatory step for HFS-induced plasticity phenomena (LTP). In contrast to Gys1 and Pygb the level of mRNA for Glns was markedly higher after LTP induction than upon BS. Presumably, it reflects an upregulation of Glns following a massive influx of glutamate taken up into astrocytes by excitatory amino-acid transporters (EAATs).

The largest changes in gene expression in young animals occurred in astrocytes whereas in neurons either no change was observed (*Glns, Pygb*) or increase due to basal stimulation or HFS was much smaller than in astrocytes (Figure 3). Most interestingly, in aged animals, BS and HFS stimulation enhanced the mRNAs levels both in astrocytes and neurons but, in contrast to young rats, this effect was pronounced in neurons. Noteworthy, the Pygb inhibition in slices from aged animals did not disrupt LTP maintenance (as it did in young animals) even if in both groups BAY administration resulted in down regulation of gene expression for Gys1 and Pygb (Figure 3K, 3J and Figure 3N, 3M). The molecular mechanisms underlying this cross-talk between neuronal activity and gene expression remains unclear. Among potential intracellular effectors involved in neuron-astrocyte interplay, adenylate cyclase (Adc) seems to be particularly interesting as it has been shown that glycogen breakdown and lactate release from astrocytes was stimulated by cAMP [32]. Adenylate cyclase is also known to be activated in response to elevation of bicarbonate titer during intense neural activity [32]. Since cAMP may stimulate transcription of several metabolic genes [33] thus, increased level of cAMP may be responsible for the observed elevation of the metabolic enzymes after BS and HFS. However, our finding that Pyg inhibition resulted in the attenuation of the expression of the metabolic enzymes suggests that other molecules, which must come from glycogen-derived glucosyl units, are also engaged in regulation of the expression. It cannot be also excluded that the expression of Gys1 and Pyg in astrocytes is activated by unknown molecule released from neurons as a feedback to increased lactate release from astrocytes.

Our morphological studies showed that in aged rats the laminar structure of the hippocampal formation is severely distorted and beta-amyloid plaques were present (Figure 4). These profound morphological alterations raise several possibilities regarding changes in the neuronal-astrocytic cross-talk in aged animals. It is well known that astrocytes form several contacts with neuronal cells, especially at synapses and this observation gave rise to a concept of a “tripartite synapse” [34, 35]. It is possible that such severe morphological changes in aged rats could affect this intimate astrocyte-neuronal interaction. Presumably, precise arrangement of the tripartite synapses is a prerequisite for proper functioning of astrocyte-neuron lactate shuttle. Otherwise, lactate which is not properly taken up by neurons may become toxic and in that case, blockade of glycogen phosphorylase with BAY would be beneficial to the neuronal tissue [36, 37]. In addition, impaired astrocyte-neuronal communication in aged animals is likely to result in a reprograming of gene expression in neurons resulting in a more abundant mRNA expression for key metabolic enzymes considered here which normally (in young animals) are preferentially expressed in astrocytes.

In conclusion, we provide the first evidence that LTP in the hippocampal Sch-CA1 pathway is differentially modulated by astrocytic metabolism in young and aging animals and that the underlying mechanisms involve altered gene expression of key metabolic enzymes, pharmacological sensitivity and spine geometry.

## MATERIALS AND METHODS

### Hippocampal slice preparation and electrophysiology

Experiments were performed on male Wistar rats divided into two age groups: young (P 27 - 35) and aged (P 550 – 700). Animals were anesthetized with isoflurane and decapitated. All procedures were approved by Local Ethical Commission and an effort was made to minimize the number of animals used for experiments. The brain was quickly removed and transverse slices (350 μm thick) were cut with a vibratome (Leica VT1200S, Germany) in ice cold buffer containing (in mM): sucrose 75, NaCl 87, KCl 2.5, NaH_2_PO_4_ 1.25, NaHCO_3_ 25, CaCl_2_ 0.5, MgSO_4_ 7, and glucose 25, oxygenated by saturation with carbogen (95 % O_2_, 5 % CO_2_), pH 7.4. Slices were transferred to a chamber containing the same carbogen saturated solution and kept at 32°C for 15 min. Slices were then kept in a holding chamber at room temperature in an oxygenated artificial cerebrospinal fluid (ACSF) containing (in mM): NaCl 125, NaHCO_3_ 25, KCl 3, NaH_2_PO_4_ 1.25, CaCl_2_ 2.5, MgSO_4_ 1.2, glucose 10, pH 7.4 for at least 1 h. The same ACSF was used in electrophysiological experiments and the preparation was continuously perfused at 9 ml / min. All solutions were continuously bubbled with 95 % O_2_ and 5 % CO_2_.

Synaptic transmission in the Schaeffer collateral-CA1 (Sch-CA1) pathway was investigated using field potential recordings as described previously [38]. Briefly, Schaffer collateral axons were stimulated with a bipolar electrode (125 um, FHC, USA), and evoked field excitatory postsynaptic potentials (fEPSPs) were recorded in stratum radiatum of CA1. Stimuli were delivered using a stimulator (A360, World Precision Instruments, USA), whereas extracellular recordings were obtained with borosilicate glass micropipettes filled with ACSF, yielding a resistance of 1 - 3 MΩ. The recorded signal was amplified (DAM80, WPI, USA), low-pass filtered at 3 kHz, sampled at 10 kHz (Digidata 1440, Molecular Devices, USA). The electrophysiology data were analyzed using pClamp 10.3 (Molecular Devices) and AxoGraphX (developed by John Clements).

Input-output curves (I-O) were obtained based on recordings of field excitatory postsynaptic potentials (fEPSP) evoked by monotonically increasing stimulus. Basal fEPSPs were recorded at stimulation intensity equal to 30 - 40 % of that giving maximum response. Synaptic long-term potentiation was evoked by high-frequency stimulation (HFS, 4 × 100 Hz, 1 s duration with 10 s intertrain intervals). Paired-pulse facilitation (PPF) is expressed as the ratio of fEPSP slope (second/first) from two responses evoked with stimuli applied with an interstimulus interval of 50 ms. The fEPSP slope was calculated as the value of the maximal slope of the descending phase between 20 % and 80 % of the negative peak response.

Depolarizing envelope area was determined as total area under 100 EPSPs within high frequency train calculated from baseline (0 mV). Positive after potential (after positivity, AFP) was measured at peak occurring at 50 - 100 ms following high-frequency trains. The stimulus intensity was identical to basal stimulation (approx. 30 - 40 % max. response).

A group of slices (labeled in the tables and figures as a “BAY”) was additionally incubated with 5 μM glycogen phosphorylase allosteric inhibitor (BAY U6751 - shortly BAY; Santa Cruz, USA) which was added to the incubation medium 30 min before LTP induction (*K_i_* = 1.6 nM, soluble in H_2_O).

### Tissue preparation

For in situ hybridization and immunohistochemistry staining, brain slices (350 μm thick) were immersed in an alcoholic fixative (1 methanol : 3 ethanol 95 %) for 20 min at 4°C and subsequently kept at −20°C. Fixed slices were washed in 99.8 % ethanol (4°C) and processed through several mixtures of ethanol and increasing concentration of low melting polyester wax (Electron Microscopy Sciences, USA) until slices were embedded in pure wax. Tissue preparations were cut into 4 μm thick sections on rotary microtome (Leica RM 2255, Germany) and mounted on glass slides (Superfrost Plus slides, Menzel Glaser, Germany) and kept at 4°C. Just before specific staining, tissue sections were dewaxed in 96 % ethanol (2 × 5 min) and 99.8 % ethanol (10 min) and rehydrated directly with phosphate saline buffer (PBS).

For morphological analysis of dendritic spines (Diolistic method) brain slices were fixed with 4 % paraformaldehyde in PBS (30 min, 4°C) and kept in cold PBS until subsequent use.

### Fluorescence *in situ* hybridization (FISH) and immunofluorescence staining (IF)

Dewaxed and rehydrated 4 μm thick sections were fixed with 4 % PFA / PBS (10 min, 4°C). Then, to localize mRNAs the slides were washed with sodium citrate buffer (SSC: 150 mM NaCl, 15 mM sodium citrate; pH 7.0) and incubated with hybridization mixture contained 1 μM specific Cy3-labeled oligonucleotide to mRNA identification, for 3 h at 45°C (5 % dextran sulfate sodium salt, 50 % formamide, 0.1 % BSA, 0.1 % Ficoll-400, 5 mM EDTA, 0.1 % polyvinylpyrrolidone, 50 μg/ml yeast tRNA, 200 μg/ml DNA from salmon testes, 750 mM NaCl, 75 mM sodium citrate; pH 7.0). List of 5′-end Cy3-labeled oligonucleotides complementary to studied rat mRNA sequences (Table 1) were synthesized by Sigma-Life Science. The FISH reaction was stopped by washing with 2 x SSC buffer (3 × 10 min, 45°C).

In order to distinguish astrocytes from neurons, the astrocyte (GFAP) and neuron (MAP2) markers were localized using immunofluorescence staining directly after FISH. For this purpose, slices were incubated for 30 min at 37°C with primary antibodies diluted in 2 x SSC buffer (anti-GFAP, 1:500, Sigma-Aldrich, cat nr G3893; anti-MAP-2, 1:250, Sigma, cat. nr M4403,) and washed with 0.05 % Tween-20/2 x SSC (1 × 5 min). Subsequently, slices were incubated for 30 min at 37°C with secondary antibodies diluted in 2 x SSC buffer (AlexaFluor 633 goat anti-mouse, 1:500, Invitrogen), and washed again. Finally, the slices were stained DAPI (Sigma) to visualize the cell nuclei and mounted in Fluoroshield. In control reaction, the oligonucleotide probes were omitted. All solutions were prepared using DEPC-water and with RNase free equipment.

Particular care was taken that sample pairs of unstimulated control (C), basal stimulated control (CS, we used basal stimulation of 0.1Hz lasting 90 minutes), HFS stimulated slices (LTP, lasting 90 minutes) as well as LTP induced slices in BAY inhibitor presence (labeled in the tables and figures as a “LTP BAY”), were from the same animal and were processed simultaneously and subsequently imaged with identical acquisition parameters (i.e. camera gain, exposition time). We tested brain slices of 4 animals for each age and treatment group. Average number for analyzed cells in probe was *n* = 2282 and *n* = 1049 for astrocytes and neurons respectively. The relative fluorescence was normalized as a percentage of initial mRNA level defined for unstimulated control samples (C).

### Morphological analysis of dendritic spines

Random dendrite labelling with a gene gun, using 1.6 mm tungsten particles (BioRad) coated with propelled lipophilic fluorescent dye (DiI; Invitrogen), was performed for the dendritic spine morphological analysis. Images of secondary apical dendrites located at 50 μm–200 μm from the cell soma of the CA1 field of hippocampus were analyzed. The purpose of this restriction was to eliminate possible systematic differences in spine morphologies that are due to the location of spines on dendrite with different ranks. Images were acquired under 561 nm fluorescent illumination using a confocal microscope (40 x objective) at a pixel resolution of 1024 × 1024 with a 3.33 zoom, which resulted in a 72 nm pixel size. Spines were measured and analyzed using semi-automatic, custom written software, Spine Magic [39]. We determined a scale-free parameter, the length-to-width ratio (i.e. the length divided by the width), which reflects the spine shape. We analyzed spine morphology of young (1 month) and aged (20-22-months) rats. 3 young and 4 aged rats were analyzed. The hippocampal slices of each rat were divided into ten experimental groups (Table 2). From each of the group, at least 300 spines were analyzed. Additionally, control groups were analyzed: C, C BAY, where hippocampal slices were fixed after brain preparation, without any exogenous stimulation.

### Immunohistochemistry (IHC)

Tissue sections were dewaxed in alcohol (96 %, 100 %) in 37°C and fixed in paraformaldehyde (10 min, 4°C). The IHC reactions were performed in the Autostainer Link 48 (Dako, Glostrup, Denmark) using an EnVision FLEX visualization system (Dako). Endogenous peroxidase was blocked by incubating sections in 3 % hydrogen peroxide. For detection beta-amyloid sections were incubated with the primary antibody diluted at 1 : 400 (monoclonal mouse antibody; Convance, cat. no SIG 39320-1000) for 20 min at room temperature. Secondary goat antibody (EnVision/HRP; Dako) were linked to horseradish peroxidase. The substrate for the reaction was DAB (3,3′- diaminobenzidine tetrachlorohydrate). The slices were counterstained with hematoxylin and coversliped with Mounting Medium (Dako, Denmark). Sections immunostained in the absence of primary antibody were used as negative controls.

### Image acquisition

Wide-field FISH+IF fluorescence was acquired with fluorescence microscope Olympus BX51 combined with Olympus DP72 camera. For each slice pictures were analyzed for: (1) number of nuclei (DAPI at 430 nm), (2) relative fluorescence for mRNA concentration (Cy3 at 540 nm) and (3) for brain cell distinction between neurons and astrocytes (GFAP/MAP-Alexa fluor at 633 nm). For IHC image acquisition we used VIS microscope Olympus BX41 combined with Olympus ColorView III camera. For each hippocampi a series of pictures with highest resolution was acquired (40 x objective at a pixel resolution of 4140 × 3096).

### Image analysis

The image analysis was performed in the following steps. First, areas of 6 - 8 adjacent visual fields (approximately 200 × 180 μm each) for CA1 regions were analyzed within each thin (4 μm) hippocampal section. To build proper statistics, the measurements were repeated for 4 independent hippocampal preparations representing the same type of electrophysiological experiment (4 x C, 4 x CS, 4 x LTP, 4 x LTP BAY). Then Cy3 fluorescence values within each group were pooled, providing a final relative fluorescence value to create the histogram curves. Such analysis was performed in hippocampal sections stained against microtubule associated protein 2 (MAP-2), a neuronal marker. In our hands, MAP-2 antibody stained both soma and dendrites of rat hippocampal neurons and therefore it was used as a reference of neuronal structure (Figure 3A-3D). Parallel hippocampal sections were stained additionally against GFAP protein, which enabled us to analyze astrocyte soma with astrocyte tabs (Figure 3E-3H). The analysis of mean Cy3 fluorescence was performed within regions of interest (ROIs) which were exclusively in MAP-2 positive cells (i.e. neurons, Figure 3D) of CA1 *stratum pyramidale*, within GFAP positive cells (i.e. astrocyte) in the same region and within GFAP positive cells in CA1 *stratum radiatum* (Figure 3H). Image analysis was performed using Olympus Cell^F software. Fluorescence intensity for Cy3 staining, corresponding to mRNA level in neurons or astrocytes was corrected by subtracting the background fluorescence level.

### Statistical analysis

Data were analyzed using unpaired t-test or ANOVA. Signiﬁcance in the ANOVA was followed by the Holm-Sidak post-hoc test. The statistical analyses were performed using SigmaStat 3.1 software (Systat Software). Data are expressed as mean ± SEM with signiﬁcance levels of *p ≤ 0.05, **p ≤ 0.01, ***p ≤ 0.005. In electrophysiology results, *n* refers to the number of individual slices from different animals. In FISH and IF *n* refers to number of analyzed cells (astrocytes or neurons).

For dendrite spine morphology analysis, the data are expressed as mean +/− standard error of the mean (SEM). Datasets were tested using the two-tailed Student's t-test and if the number of groups were larger than two, one-way ANOVA was used. For statistical analysis of cumulative distribution plot Kolmogorov-Smirnov test were employed. Statistical analyses were performed using Origin 8 software (Origin Lab Corporation).
